# Early Origins of Psoriatic Arthritis: Clinical, Genetic and Molecular Biomarkers of Progression From Psoriasis to Psoriatic Arthritis

**DOI:** 10.3389/fmed.2021.723944

**Published:** 2021-08-18

**Authors:** Stephen R. Pennington, Oliver FitzGerald

**Affiliations:** Conway Institute for Biomolecular Research, School of Medicine, University College Dublin, Dublin, Ireland

**Keywords:** psoriatic arthritis, genomics, proteomics, biomarkers, risk factors, psoriasis

## Abstract

Greater than 90% of patients with psoriatic arthritis (PsA) first develop their arthritis on a background of known psoriasis (Pso). Thus, having skin/nail Pso certainly is an important risk factor for PsA but as PsA develops in <30% of those affected with Pso, the presence of Pso alone is insufficient as a means of identifying which patients with Pso will develop PsA. It is hoped that with further molecular assessment of Pso patients who do not have any evidence of inflammatory musculoskeletal disease compared to those with early PsA features, that the “at risk” profile of Pso patients destined to develop PsA can be refined such that disease prevention studies can be designed and a new era of treatment for PsA can emerge. In this article, the early stages in the development of PsA are outlined and what is currently known about clinical features, genetic factors and soluble or tissue biomarkers associated with the development of PsA in patients with Pso is reviewed in detail. Finally, proposals are outlined regarding the approaches required in order to address this important research area.

## Introduction

Psoriatic arthritis (PsA) is a chronic immune-mediated condition that results in systemic musculoskeletal (MSK) inflammation and consequent impairment in a person's quality of life and of function (1). Most patients develop PsA on a background of long-established psoriasis (Pso) although the period of Pso may in some patients be very short and in under 10% of people, the musculoskeletal manifestations of PsA predate the development of skin or nail Pso (2). The diagnosis of PsA is clinical and diagnostic accuracy often depends on the knowledge and skills of the treating physician. There is no diagnostic test for PsA and as a consequence diagnosis is frequently delayed with studies showing that a delay in diagnosis of >6 months contributes to poor outcomes including radiographic outcomes (3). The challenge is to diagnose PsA early and if possible at the time of the first appearance of any MSK symptoms, when treatment intervention is likely to have the most significant impact on preventing or limiting disease progression. A greater challenge though is to accurately identify Pso patients at significant risk of developing PsA when treatment intervention might prevent PsA from occurring at all.

Here, the evidence for the various stages in the development of PsA will be reviewed. Clinical and genetic factors identified as indicating greater risk of PsA development in Pso patients will then be summarised and subsequently tissue and soluble biomarkers which have found some utility in single-centre studies will be introduced. To conclude, we will propose a systematic, unbiased approach to the identification, verification and validation of biomarkers associated with increased risk of development of PsA in patients with Pso.

## When Does PsA Begin?

For a patient with inflammatory MSK symptoms or findings associated with skin and/or nail Pso, the experience of having PsA really starts with a diagnosis. There are however no diagnostic criteria or diagnostic tests for PsA and the diagnosis often depends on the clinician's knowledge and experience. A diagnosis of PsA is most commonly reached when there is clear evidence of inflammatory MSK disease, in the presence of Pso and in the absence of rheumatoid factor (RF) and anti-Cyclic Citrullinated Peptide (anti-CCP) antibodies. There are several pitfalls in this approach including the transient nature of symptoms and findings in early disease, the difficulty in separating inflammatory from degenerative findings or from fibromyalgia, the absence of Pso in 10% of patients and the presence of usually low titre RF or anti-CCP in ~10% of patients with clear PsA features. These diagnostic confounders add to diagnostic delay and contribute to the poor outcome experienced by patients when diagnosis is delayed by 6 months or more (3).

Furthermore, in the setting of early, undifferentiated inflammatory arthritis, making a diagnosis of PsA can often be challenging because:

- Symptoms and signs of PsA in early disease may fluctuate;
- There is commonly under-recognition of inflammation with imaging studies (US/MRI/PET-CT) often identifying areas of sub-clinical synovio-entheseal involvement;
- Skin and nail psoriasis may be absent in up to 10% of patients;
- RF and/or ACPA may be positive in up to 10% of patients; and
- Inflammatory markers (C-reactive protein and Erythrocyte Sedimentation Rate) are commonly normal or only minimally elevated.

**Figure 1:**
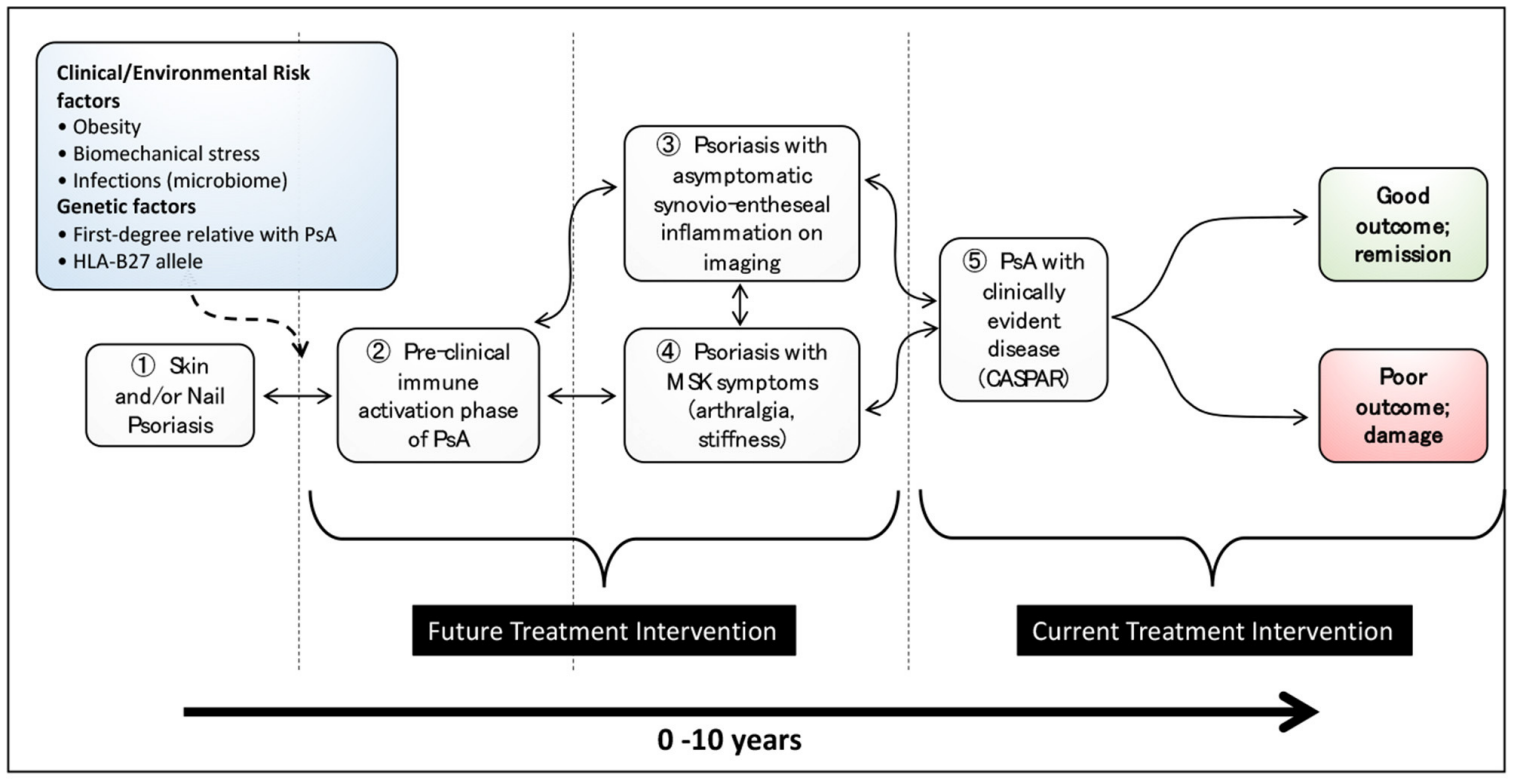
Stages in the development of psoriatic arthritis in patients with psoriasis (from Nature Reviews Disease Primer on Psoriatic Arthritis; accepted, in editorial review where figure has been re-drawn).

What is becoming clear over recent years is that PsA likely begins in many if not most patients at a much earlier point, perhaps years before the eventual diagnosis is made. A recent consensus study (4) defined the terminology to be used before PsA is diagnosed and proposed 3 stages: (1) individuals with Pso at increased risk of PsA; (2) individuals with Pso and asymptomatic synovio-entheseal imaging abnormalities; and (3) individuals with Pso and MSK symptoms not explained by other diagnoses. The evidence for these stages will be reviewed below. Other disease stages may also exist with an immune activation stage being proposed where there may be asymptomatic molecular evidence of MSK involvement without inflammatory imaging features (5). The stages in the development in PsA are illustrated in Figure 1.

Current research strongly suggests that the development of PsA is the result of interplay between environmental factors, an individual's phenotype and genotype and both the adaptive and innate immune system. If the early stages of immune activation can be addressed before disease becomes established, it is possible that some of the stages shown in Figure 1 may be reversible, as indicated by the bidirectional arrows. At present, treatment is focused on those patients who receive a PsA diagnosis (stage Ⓢ in Figure 1) and have ongoing inflammatory disease and evidence of radiographic damage. Future treatment intervention needs to focus on earlier stages of disease so as to limit poor long-term outcomes and possibly prevent the development of PsA. Improving our knowledge of the molecular basis of these stages and the transitions between them will enable us to have a deeper understanding of the progression of psoriasis to PsA. In turn, the development of stage-specific biomarker signatures will undoubtedly move the treatment paradigm from a focus on established and progressive disease to one focused on much earlier intervention and possibly disease prevention. Finally, future large prospective studies of patients with Pso will be needed to understand these disease stages further and in particular to define the optimal point where disease prevention studies should be targeted.

### Pso at Increased Risk of PsA; Clinical Predictors or Risk Factors

Certain clinical features of Pso such as nail pitting, psoriasis location and the extent/severity of disease might help identify patients with early PsA or those patients with skin/nail Pso only most likely to develop PsA. Whether these variables represent predictors or risk factors is a matter of active investigation (5).

A longitudinal, retrospective, population-based cohort study of subjects with Pso performed by Wilson et al. determined the incidence and disease-specific predictors of clinically recognised PsA in patients with Pso where patients with clinically recognised PsA (determined from a review of the case records) were excluded from the analysis (6). The psoriasis incidence cohort comprised of 1,593 subjects. Over 20,936 person-years of follow-up, 57 patients were clinically identified with new-onset PsA, with a cumulative incidence of 1.7, 3.1, and 5.1% at 5-, 10-, and 20-years following psoriasis incidence, respectively. Psoriasis features associated with a higher risk of PsA were scalp lesions, nail dystrophy and intergluteal/perianal lesions (6).

In a large, prospective, single-centre cohort study involving Pso patients who were screened annually for arthritis, Eder and colleagues investigated the incidence of PsA in patients with cutaneous psoriasis (PsC), defined as cutaneous psoriasis patients where the presence of PsA has been excluded following full rheumatologic assessment (7). To identify risk factors associated with the development of PsA, information was collected about lifestyle habits, comorbidities, psoriasis activity and medications. The data obtained from the 464 patients who were followed up for 8 years were analysed. A total of 51 patients developed PsA during the 8 years following enrolment. The annual incidence rate of PsA was 2.7 cases per 100 PsC patients. In multivariate analysis, the following baseline variables were associated with the development of PsA: severe psoriasis, low level of education and use of retinoid medications. In multivariate models with time-dependent variables, psoriatic nail pitting and uveitis were associated with the development of PsA (7). The prospective, longitudinal nature of this study and the fact that each Pso patient was examined annually by an experienced rheumatologist add strength to these study findings.

In both the studies described above, the findings that psoriatic nail pitting and nail dystrophy are associated with the development of PsA could be explained by the fact that psoriatic nail disease is associated with enthesitis. Inflammation of the enthesis possibly precedes joint involvement, and it may be the reason why nail disease precedes PsA development. Therefore, nail disease may be a marker of increased “immunoreactivity,” which could in turn lead to the development of PsA (8).

In an interesting approach from the UK PROMPT study group, a Bayesian statistical approach was used to identify MSK symptoms influencing the risk of developing PsA in >90,000 people with Pso (9). In an analysis of >I million MSK symptoms, gender, BMI, arthralgia, finger pain, fatigue, hand pain, hip pain, knee pain, swelling, back pain and myalgia were related to PsA development. The best Bayesian network achieved an area under the receiver operating characteristic curve of 0.73.

There are a number of non-psoriasis related features, possibly modifiable, which may also increase the risk of PsA development. Several studies have shown that obese patients with psoriasis and obese patients in general have an increased risk for the development of PsA (5). In a UK population-based study of > 75,000 patients with Pso, the risk ratio (RR) for developing PsA was 1.09 (0.93–1.28) for Body Mass Index (BMIs) from 25.0 to 29.9 kg/m^2^, 1.22 (1.02–1.47) for BMIs from 30.0 to 34.9 kg/m^2^, and 1.48 (1.20–1.81) for BMIs C 35.0 kg/m^2^ (10). A more recent cohort study of 90,189 incident cases of Pso has confirmed these findings and has also demonstrated that reducing BMI over a 10-year period was associated with a reduction in the risk of developing PsA compared with BMI remaining constant over the same period (11). In the same study, increased risks of developing PsA were associated with moderate drinking but not with former or heavy drinking or with current or past smoking status. These latter findings are in general consistent with previous literature on the topic though the finding of a lack of association with smoking is quite different from the literature in rheumatoid arthritis (RA) (12).

### Pso at Increased Risk of PsA; Genetic Markers Distinguishing Pso From PsA

To date, many efforts have been made to characterise the genetics of PsA with early work yielding divergent results. The interpretation of results was complicated by the imprecise serologic definition of HLA alleles and the lack of rigorous PsA classification criteria (13). Two studies from Canada and Ireland were then published that used newer DNA-based methods of typing HLA alleles and the ClASsification of Psoriatic ARthritis Criteria (CASPAR) criteria for case ascertainment (13, 14). These studies similarly showed that PsA was indeed genetically heterogeneous as not only were some PsA patients HLA-B^*^27 and others HLA-C^*^06:02, but additional alleles were found at significantly increased frequencies in these well-classified patient cohorts.

Intriguingly, in further analysis of the Irish cohort, C^*^06:02, was found, as anticipated, in 58% of those with cutaneous Pso as compared to 28% of those with PsA (13). This difference was so statistically significant that the hypothesis that the Pso phenotype is genetically homogeneous could be rejected. Eighteen percentage of PsA cases were typed as having B^*^27, while the frequency of B^*^27 in cutaneous Pso was not significantly elevated as compared to controls. Interestingly, B^*^08 was the major allele in the PsA cohort, accounting for 37.3% of all cases, vs. 26.1 in controls and significantly reduced in frequency to 15.6% in those with Pso only. The findings in the Canadian study were broadly similar (14). Lastly, significant associations of PsA with either B^*^39 or B^*^38 were found in 15% of PsA cases, bringing the total number of different allelic associations to five, underlining the genetic heterogeneity of PsA.

Multiple non-HLA genes have also been identified as being associated with possible risk of PsA in patients with Pso, including polymorphisms within relevant inflammatory pathways. However, their effect size tends to be small (~1.2–1.3), and this limits their utility (15). It is important to note that all genes that have been identified as being more or less frequent in PsA may not directly be a risk factor, as in causative, for the disease but may simply be associated. In other words, while identified genes may indeed play a role in PsA pathogenesis, they can be associated with development of PsA without having a defined pathogenetic role. It is certainly possible that knowledge of risk of PsA will increase with greater awareness of multiple genotypes and their interactions. It is for this reason that the study of much larger case series of carefully ascertained PsA patients across multiple clinics and in different geographic regions is necessary so as to obtain statistically significant numbers of different gene combinations. It is evident that studies of genetic markers show there is little to differentiate Pso and PsA although some studies have revealed changes in variants of the IL-23 receptor in a subsets of T lymphocytes and CD8 T cells (16, 17). The study by Bowes et al. unsurprisingly demonstrated substantial allele sharing between those with Pso and those with PsA but their results are such that they provide compelling support for the existence of PsA-specific loci (16). While we have gained knowledge relating to the genetic contributions to PsA, thus far, routine genetic screening is not yet recommended owing to the low prevalence and lack of sufficient evidence of improvement in the diagnosis of PsA (15).

### Pso at Increased Risk of PsA; Epigenetic Markers Distinguishing Pso From PsA

The possible role of epigenetic factors which might identify Pso patients at risk of developing PsA has been nicely reviewed in 2 recent articles (18, 19). There is early evidence for changes in methylation levels of some genes in patients with skin and joint disease and in patients with joint disease (20) with IL22 being proposed as a possible germ line risk factor for PsA. Further studies would be required to confirm these findings but results are of interest given the known expression of IL22 in both the skin, at the enthesis and in synovial fluid (20). Well-designed epigenetic studies, including a focus on DNA methylation studies, histone modification studies and epigenome-wide association studies (EWAS), are required to examine whether epigenetic markers will have a role in distinguishing Pso patients at risk for progression to PsA.

### Pso and Asymptomatic Synovio-Entheseal Imaging Abnormalities

Imaging, notably ultrasound (US), magnetic resonance imaging (MRI), high-resolution peripheral quantitative computed tomography (HR-pQCT) and positron emission tomography-computerised tomography PET-CT, might possibly be used as tools to identify asymptomatic Pso patients who will develop PsA. That asymptomatic Pso patients have US-detected abnormalities has been known for some time and is nicely reviewed by Zabotti et al. (21). Gisondi et al. undertook ultrasonographic evaluation of entheseal sites commonly involved in PsA in 30 Pso patients and 30 controls, scoring their findings according to the Glasgow Ultrasound Enthesitis Scoring System (GUESS) (22). The mean GUESS score and in particular tendon thickness was significantly higher in psoriasis patients vs. controls. Interestingly, GUESS score correlated with age and body mass index raising concerns about the specificity of the findings. The same authors then investigated whether subclinical enthesopathy in patients with Pso predicted the future development of PsA (23). Seven of 30 Pso patients followed for a mean of 3.5 years had developed PsA but the difference in GUESS scores compared to those who did not develop PsA did not reach statistical significance. In a similar study by Elnady et al. in 109 psoriasis patients, there was a statistically significant higher association of baseline enthesitis power doppler and grey scale US scores and of synovitis scores in psoriasis patients who developed PsA (24). In a further study, the ultrasonographic characterisation of arthralgia was examined in Pso patients with and without arthralgia and in normal controls (25). Interestingly, only the presence of tenosynovitis on US was significantly associated with arthralgia. Finally, taking it one step further, Savage and colleagues treated 23 Pso patients whose ultrasound showed inflammatory changes with interleukin-12 (IL-12)/IL-23 inhibition (ustekinumab) for 52 weeks (26). With treatment, the mean inflammation scores but not entheseal structural abnormalities decreased significantly with suppression maintained through week 52. Whether this translates into prevention of the development of PsA remains to be proven.

The findings of subclinical synovitis and enthesitis on MRI in Pso patients without evidence of PsA was also described some years ago (27, 28). Some longitudinal data was provided by Faustini et al. who provided short-term follow-up information (29). At baseline, 47% of patients with psoriasis showed at least one inflammatory lesion on MRI. The risk for developing PsA was as high as 60% if patients had subclinical synovitis and symptoms related to arthralgia, but only 13% if patients had normal MRIs and did not report arthralgia. The authors appear to relate arthralgia to joint tenderness which may not necessarily be the case as patients may experience arthralgia without joint tenderness, with the reverse being true as well. In a follow-up study, 20 patients with Pso but not PsA were selected for treatment with secukinumab (30). Patients had to have nail or scalp involvement or a high psoriasis area severity index (PASI) (>6) as well as inflammatory or erosive changes on MRI or CT. Skin disease and arthralgia symptoms significantly improved with treatment at 24 weeks. Total PsA MRI Score (PsAMRIS) (*p* = 0.005) and synovitis sub-score (*p* = 0.008) also significantly improved providing some rationale for early intervention studies targeting prevention of the development of PsA in Pso patients considered at high-risk. With the high percentage of at risk Pso having MRI abnormalities, the inclusion of a placebo arm in study design is going to be important so as to study the natural history of these MRI abnormalities.

In a study by Simon et al., the presence or absence of structural entheseal lesions was assessed by high-resolution peripheral quantitative computed tomography (HR-pQCT) in 114 patients with psoriasis without clinical evidence of MSK involvement (31). Results showed that while arthralgia at baseline increased risk of progression to PsA, the presence of structural entheseal lesions further enhanced the risk for progression to PsA both in the absence and presence of arthralgia, with the highest progression rate in those subjects with both arthralgia and entheseal structural lesions.

The literature regarding the ability of FDG-PET-CT to detect subclinical inflammation is more recent. Takata et al. assessed 18 Pso patients and 28 PsA controls and found evidence of asymptomatic enthesitis in 6/18 patients (32). To date, prospective longitudinal and treatment intervention studies have not been reported.

### Pso and MSK Symptoms Not Explained by Other Diagnoses

In the Toronto prospective Pso cohort referred to earlier, the authors further assessed whether the presence of non-specific MSK symptoms predicted the development of PsA (33). They found that patients who present with high levels of fatigue, pain, and stiffness and those who show gradual worsening of these symptoms were much more likely to subsequently develop PsA. It would appear that many patients subsequently diagnosed with PsA experience a period of time, sometimes lasting many months, where they have these symptoms but with little to find clinically to confirm MSK inflammation or any other cause for the symptoms. Interestingly, the presence of arthralgia in women but not in men was predictive of PsA development. This stage of disease requires further study, but it may well be that this is an important prodromal phase of PsA in many patients where targeted intervention might prevent further disease progression.

## Cellular and Tissue Markers of PsA in Patients with Pso

An early study of skin from PsA patients (*n* = 15) vs. Pso patients (*n* = 5) and normal control skin (*n* = 4) identified increased numbers of CD45Ro T-cells, greater vascularity, the presence of B-cells, and increased numbers of DR +epidermal cells as markers for arthritis in patients with Pso (34). While these findings have not been replicated, a more recent study by Leijten et al. identified CCR10+ CD8+ T cells as being enriched in PsA peripheral blood (35). CCR10+ CD8+ T cells were detected under inflammatory and homeostatic conditions in Pso skin but were not enriched in synovial fluid. The authors suggest that aberrances in cutaneous tissue homeostasis and the expression of tissue-resident memory CD8+ T cells in both skin and peripheral blood may contribute to the development of PsA.

Studies of peripheral blood mononuclear cells from Pso patients with PsA have revealed higher expression of genes associated with an IFN signature in their monocytes and CD3+ T cells from patients with PsA showed increased secretion of IL-2 following stimulation with anti-CD3 (36, 37).

## Soluble Markers Differentiating Pso vs. PsA

As previously noted, detecting PsA in clinical practise is time-consuming and requires a blend of physician experience and radiographic imaging – a challenging situation compounded by the lack of serum protein diagnostic biomarkers. This lack of well-validated diagnostic tools means PsA can be often misdiagnosed or underdiagnosed.

Despite the clear need for better (even some) diagnostic tools there have been relatively few biomarker studies undertaken using well clinically phenotyped cohorts of Pso and PsA patients Some studies have led to the identification of candidate biomarkers that may discriminate Pso from PsA and such biomarkers include CRP or inflammatory cytokines such as IL-6, IL-23 and TNF-a, markers of bone or cartilage damage and adipokines (38–47). A pilot study found that hsCRP, OPG, MMP-3 and the CPII:C2C ratio are biomarkers for PsA in patients with Pso. The data was generated from a small group of carefully selected and phenotyped patients with Pso, PsA and healthy controls and revealed that there are candidate soluble biomarkers that can distinguish patients with PsA from those with Pso alone (38). In a more recent study, soluble proteins were examined in Pso patients without arthritis who converted to PsA (converters) and Pso patients who did not develop PsA (non-converters) from a longitudinally followed prospective cohort. Baseline serum concentrations of CXCL10 were measured by Luminex assay in 46 converters and 45 non-converters. The level of CXCL10 was significantly higher in converters compared to non-converters. CXCL10 was associated with conversion status after adjustment for age, sex, duration of psoriasis, and duration of follow-up (48). Further, in a case-control study by Cretu et al., it was reported that serum levels of ITGβ5, M2BP, and CRP were independently associated with PsA compared to Pso. The combination of these three markers could differentiate PsA from Pso (49).

Based on a growing panel of candidate biomarkers, Rahmati et al., undertook a computational approach to identify nine alternative signatures obtained by combining clinical and protein markers to improve discrimination between PsA and Pso (50). 192 PsA and 191 Pso patients, where inflammatory MSK involvement had been excluded, had serum samples taken. Serum samples were tested for sixteen protein markers and this data integrated with four clinical features. The most significant factors influencing classifier performance were nail psoriasis and CRP, followed by SPP1. CRP, DEFA1, LEP, SOST, SPP1, TFCP2 CPII, TNFRSF11B, and nail psoriasis offered the most substantial discrimination between PsA and Pso, but it was noted that further research is required to validate these results (50). Other recent studies have shown that patients with PsA have higher levels of autoantibodies directed against carbamylated LL37 and ADAMTSL5, both of which are suggested auto-antigens in Pso (51, 52).

Despite all these initial studies suggesting there may be different levels of serum proteins in patients with Pso compared to those with PsA, a broad screen of 951 serum proteins using an affinity-based proteomic platform applied to 18 Pso patient samples compared to 20 PsA patient samples concluded that these patients shared a broadly similar serum proteomic signature (53). In this important study, samples were taken from patients at an early stage of PsA and were matched with severity of skin disease to the Pso patients. Interestingly, proteins that correlate with specific clinical features such as number of swollen joints (PsA) and PASI score (Pso) were identified. The former was positively correlated with ICAM-1 and CCL-8 while the later with PI3 and IL-17 receptor A. The authors suggested that future studies to identify factors which drive progression to PsA in patients with Pso might more productively focus on skin and synovial tissues.

Indeed, in a study conducted by Cretu et al. (54) skin biopsies were taken from involved and uninvolved skin of 10 PsA and ten age/gender matched Pso patients. The proteomes of pooled skin samples were characterised in an unbiased manner using liquid chromatography coupled to tandem mass spectrometry. When comparing PsA-derived skin to PsC-derived skin, 47 proteins were found to be elevated. To quantify these possible PsA markers in individual skin samples, a targeted multiple reaction monitoring approach was used, and eight markers were verified in an independent sample set. Subsequently, ITGB5 and periostin were measured in serum samples from 33 PsA and 15 Pso patients using enzyme-linked immunosorbent assays. ITGB5 was significantly elevated in PsA serum. ITGB5 and periostin correlated significantly in both patient groups, which suggests that these two biomarkers may be used as part of a panel of markers to screen for PsA in Pso patients (54).

This summary of selected protein biomarker studies suggests that there remains an important opportunity to continue to develop protein biomarkers that might have clinical utility in the management of patients with Pso who may progress to PsA. These future studies will undoubtedly benefit from incorporation of:

i) large well-characterised cohorts of patients with longitudinal sampling;
ii) the application of a range of proteomic strategies including unbiased LC-MS approaches as well broad panel screens for candidate discovery;
iii) continued use of targeted proteomic methods for evaluation of multiple protein candidate;
iv) application of existing and more sophisticated multivariate statistical methods including machine learning and random forest analysis;
v) combination of clinical, genetic and soluble biomarker measurement in the multivariate statistical analysis.

To date, studies of the risk factors and mechanisms underpinning progression of Pso to PsA have struggled to make progress due both to the relatively small data sets available at individual centres and also to the considerable heterogeneity of disease presentation and progression. While exciting recent work has highlighted biomarkers of interest (including those presented above), there is an urgent need for studies that include sufficiently large cohorts of patients.

Advancing from biomarker identification to validation and ultimately to use in the routine clinical setting presents its significant challenges but we suggest that this is most likely to be achieved successfully if future studies are undertaken using collaborative multi-group, multi-cohort approaches and multi-analyte investigations incorporating relevant quality controls focussed on the development of new diagnostic tools.

## Summary and Conclusion

Confirming the features characteristic of patients with psoriasis at increased risk of PsA transition and validating proteins that may distinguish PsA from PsC should help narrow a fundamental research gap as the PsA field matures. Several conditions are essential to successfully conduct investigations to identify transition risk, predict progression, and prevent PsA. The longitudinal characterisation of a large cohort of psoriasis patients at increased risk of PsA transition; close cooperation between rheumatologists and dermatologists with experience in various psoriatic disease subareas; and the participation of researchers as well as patient research partners (5). The organisation and amalgamation of those efforts could create a prediction model for PsA that would include clinical features (such as nail disease and extent of psoriasis) and serum protein biomarkers. Through prospective studies, the biomarker-based prediction model could be validated in early and established disease allowing for the determination of negative and positive predictive values, clinical utility, and cost-effectiveness (49).

## Author Contributions

All authors listed have made a substantial, direct and intellectual contribution to the work, and approved it for publication.

## Conflict of Interest

The authors declare that the research was conducted in the absence of any commercial or financial relationships that could be construed as a potential conflict of interest.

## Publisher's Note

All claims expressed in this article are solely those of the authors and do not necessarily represent those of their affiliated organizations, or those of the publisher, the editors and the reviewers. Any product that may be evaluated in this article, or claim that may be made by its manufacturer, is not guaranteed or endorsed by the publisher.
